# High-dimensional solid-state NMR facilitated by transverse-mixing optimal control

**DOI:** 10.21203/rs.3.rs-8003957/v1

**Published:** 2025-11-20

**Authors:** Alexander Klein, Jan Blahut, Suresh K. Vasa, Dmitrii Blohkin, Zdeněk Tošner, Rasmus Linser

**Affiliations:** 1 Department Chemistry and Chemical Biology, TU Dortmund University, Otto-Hahn-Straße 4a, 44227 Dortmund, Germany; 2 Department of Chemistry, Faculty of Science, Charles University, Albertov 6, 12842 Prague, Czech Republic; 3 Institute of Organic Chemistry and Biochemistry of the CAS, Flemingovo nám. 2, 16610, Prague, Czech Republic

**Keywords:** NMR Spectroscopy, Sensitivity enhancement, Higher-dimensionality experiments, Resonance assignment

## Abstract

Owing to fast magic-angle spinning, solid-state nuclear magnetic resonance has evolved as a versatile method to decipher protein structure, dynamics, and chemical properties. While higher-dimensional approaches such as 4D and 5D correlation spectra are generally capable of exceeding the established molecular-weight limitations by ameliorating signal overlap, their intrinsic implications for sensitivity and measurement times required have severely limited these important prospects in practice. Here we show that the extensive use of dedicated transverse-mixing optimal control pulses (TROPs) in high-dimensional experiments to enable concomitant transfer of complex signals along complex magnetization transfer pathways can reduce the necessary measurement time by an order of magnitude. Owing to the multiplicative benefits of the enhancement for successive indirect chemical-shift dimensions, combined with non-linear benefits upon spectral reconstruction, the combination of non-uniformly sampled, higher-dimensional approaches with an extensive use of TROPs hence presents itself as transformative for the conventional limitations of solid-state NMR.

## INTRODUCTION

Solid-state NMR is used for the characterization of solid proteins, including supramolecular assemblies^1, 2^, membrane proteins^3, 4^, fibrils^5, 6, 7, 8^, micro-crystalline preparations^9, 10, 11, 12^, and protein sediments^13, 14^, for which a diverse set of atomic-resolution information is obtained. Targets are in particular those proteins that (as monomers or more often in their higher-oligomeric states) would be associated with long tumbling correlation times that compromise solution NMR assessment, whereas magnetization transfer efficiency in the solid state is irrespective of molecular weight.^15, 16, 17^ In addition to structural features, as for instance fibril topologies, membrane protein structure, or the architectures of supramolecular structures, chemical information, such as protonation states, can be obtained.^12, 17, 18^ Most importantly, in contrast to most other structural-biology techniques, solid-state NMR also grants in-depth access to motional features over a large range of timescales, most prominently including fast (ps-ns) and intermediate (μs-ms) timescale motion.^19, 20, 21, 22, 23, 24^ In order to access the different site-specific attributes, resonance assignment tends to represent a challenging overarching precondition. In particular for larger-molecular-weight targets, the respective procedures suffer from a high level of ambiguity, combined with a generally lower sensitivity of any experimental schemes due to the fewer copies of target protein in the rotor. The sensitivity problem applies in particular to those (more sophisticated) experiments that in principle would be able to overcome the ambiguity bottleneck, such as higher-dimensionality sequences^25, 26, 27, 28, 29^, direct amide-to-amide correlations (and H^α^-detected derivatives)^30, 31, 32, 33^, and sidechain-to-backbone correlations^34, 35, 36, 37^.

For solution NMR spectroscopy of small organic molecules, a common way of increasing the available signal-to-noise ratio (SNR) is the use of “sensitivity-enhanced” or “preservation of equivalent pathways” (PEP) sequences.^38^ Instead of transferring only one out of the two (real/imaginary) components of the complex signal in a given magnetization transfer step, both components are maintained and later separated via linear combinations of slightly differently recorded FIDs.^39^ However, PEP-blocks in solution double the length of the “enhanced” magnetization transfer step and hence, at the same time, significantly increase relaxation losses when *T*_2_ relaxation is non-negligeable. Accordingly, maximally a single PEP element, enhancing magnetization transfer from ^13^C or ^15^N back to protons, has generally proven to be useful, and only for very small proteins a sensitivity gain of at most ~1.4 can be obtained.^40^ Recently, the possibility of sensitivity-enhanced cross polarization (CP) transfers either through transverse-mixing optimal control pulses (TROP)^41^ or by simplified preservation of pathways (SPEPS)^42, 43^ was introduced, which transfers the PEP principles to solid-state NMR. Compared to solution, the approach is irrespective of molecular weight and can be applied with little extra burden to multiple indirect dimensions. Whereas the overall concept, including simple applications such as to hCANH and hCONH experiments^44^ has recently been demonstrated,^41, 45^ the major beneficiary of sensitivity enhancement are the current state-of-the-art approaches associated with higher spectral dimensionality, which have started to evolve as a routine tool ameliorating the ambiguity bottleneck of more complex targets in the last few years but on their own have been associated with intrinsic sensitivity limitations. The prospects of multiple sensitivity-enhanced dimensions in such approaches, however, have been inconceivable practically at this point due to significant conceptional hurdles regarding their technical implementation with regards to pulse sequence design and data processing, in particular the combination of multiple echo-antiecho (E/A) dimensions with non-uniform sampling, signal reconstruction, and water suppression.

In the following, we demonstrate the mastering of these conceptional hurdles, which together with a broad range of developed TROP pulses, has a transformative impact onto sensitivity and feasibility, with time savings of almost an order of magnitude in acquisition time being achieved, along with detailed instructions for data processing using different algorithms given in the SI and an associated data publication. Together with non-linear sensitivity enhancements and hence larger-than-expected overall benefits, the demonstrated experiments and underlying concepts highlight unprecedented prospects for solid-state NMR feasibility that are robust across different spectrometer setups and sample preparations.

## RESULTS AND DISCUSSION

### Design Principles and Processing

Pulses derived from optimal-control theory (OCT) harbor great benefits for NMR as they can be fit to solve problems for which analytically derived solutions are hard to find^46^. This has recently been demonstrated in solid-state NMR, where TROP pulses, instead of standard cross polarization schemes, can simultaneously transfer both, the real and imaginary parts of a complex FID. This ideally prevents the usual loss of a factor of 2^1/2^ in the SNR along each indirect chemical-shift dimension. However, OCT solutions are often tailored for specific purposes and can lack generality. For TROPs, this is reflected by the general necessity of specific pulse shapes for each desired transfer and spectrometer field strength, whereas for CP it is often sufficient to match a given resonance condition using standard shapes. This also holds true for the SPEPS approach, however, in slightly less severe fashion. However, TROPs can by now be created very flexibly and already exist for most commonly employed transfers over a wide range of magnetic field strengths.

**Figure 1:**
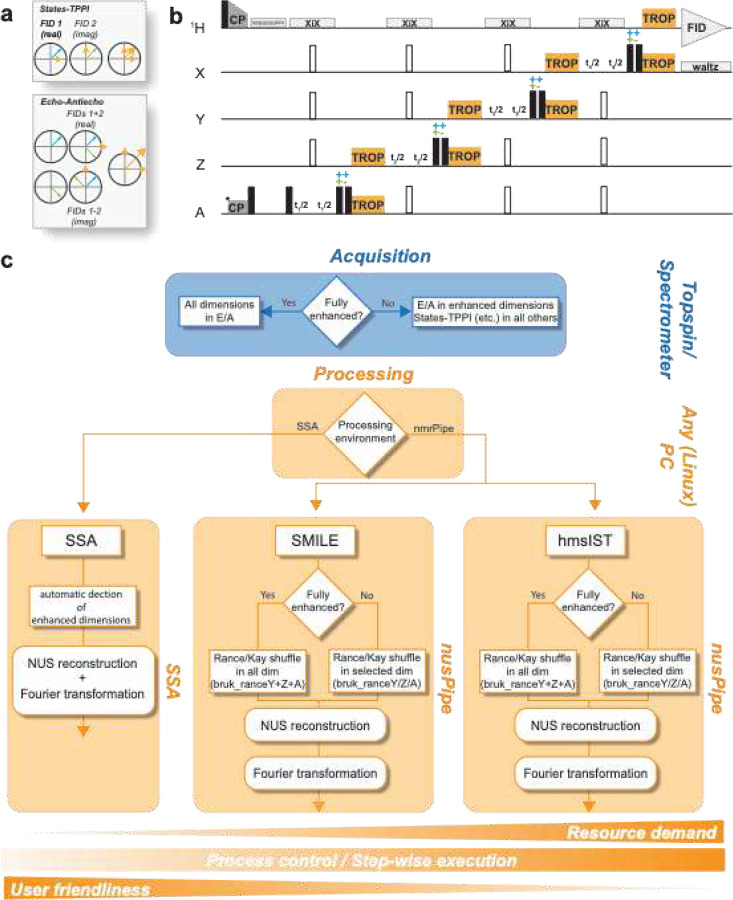
Principles of pulse sequence design and data processing. **a** Creation of hypercomplex data when either using States or States-TPPI (top) or Echo-Antiecho / Rance-Kay (bottom). The latter results in every FID to contain signal from the real and the imaginary components of the indirect dimensions, resulting in a theoretical SNR improvement of factor 2^1/2^ per indirect dimension compared to former. **b** Schematic pulse sequence for the implementation of TROP pulses to achieve sensitivity-enhancement. Essentially, any CP can be replaced by the corresponding pair of TROP pulses, flanked by two 90 ° pulses with same or opposite phase in every other increment to achieve E/A modulation. Water suppression using MISSISSIPPI cannot be applied together with TROP pulses after evolution in sensitivity-enhanced dimensions. **c** Schematic overview of suggested steps for data acquisition (blue) and data processing (orange) for a selection of reconstruction routines, depending on the level of sensitivity enhancement. In brief, SSA features a more user-friendly workflow with fewer steps and significantly lower resource demand, while changing of processing parameters often requires a repetition of the entire workflow. NMRpipe-based reconstruction requires more interaction from the user, but features step-by-step control during the process. Details are described in the SI.

Figure 1 schematically shows the design principles for incorporation of TROPs into a given, arbitrary multi-dimensional experiment. In order to grant phase-sensitive chemical-shift evolution in the framework of preserved pathways (Fig. 1a) upon exchange of CPs in a prototypical, multi-nuclear sequence against suitable TROPs, each TROP is flanked by a pair of 90° pulses (Fig. 1b). Choosing either the same or opposite phase enables inversion of one of the components of the complex signal, allowing echo-antiecho processing. Additional sensitivity-enhanced dimension can be incorporated the same way. By contrast, for such transfers that as of now cannot yet or are not intended to be enhanced, CPs are retained, with frequency discrimination in those dimensions exerted using, e.g., States or States-TPPI. In the Supporting Information, we show that States and echo-antiecho approaches can be applied in any order while maintaining the sensitivity enhancement advantage of TROP transfers. There is one conceptual intricacy on the side of the pulse sequence, however. Water suppression, as routinely implemented in current solid-state NMR methods according/similar to the MISSISSIPPI scheme^47^, only retains one of the components of magnetization. Hence, it can only be implemented *together* with a States-encoded dimension or directly after a first H/X CP when all dimensions make use of TROP transfers and no chemical-shift incrementation of the initial proton is sought.

While 3D experiments are commonly recorded by sampling the entire FID (uniform sampling; US), this is usually not feasible for experiments with four or more dimensions, as (in the resolution-limited regime) measurement times scale approximately exponentially with each added dimension. Instead, only a subset of all possible data points is recorded (non-uniform sampling; NUS) to reduce measurement time or increase resolution. In these cases, processing the obtained sensitivity-enhanced data sets usually requires processing outside the spectrometer software environment, as direct Fourier-transformation (non-uniform FT, nuFT) will result in spectra suffering from additional sampling noise introduced by the point spread function (PSF)^48^. While this has been presenting a significant hurdle for most users already, the reconciliation of echo/anti-echo with NUS reconstruction requires specific additional procedures, specifically if nmrPipe^49^ is employed as a processing environment. Reconstruction can be achieved using various algorithms, out of which we involve SSA^50^, hmsIST^51^, and SMILE^52^ here (Fig. 1c). hmsIST, SMILE, and generally all algorithms integrated in the nmrPipe environment require the recombination of the FIDs from the recorded echo-antiecho dimensions to standard complex data by an additional Rance-Kay processing step (linear combination of the two successively recorded “phases” for each combination of time increments). Only afterwards, the processing workflow can be followed as familiar for conventional data sets. If reconstruction is performed using SSA, no further modifications in the workflow are necessary, as echo-antiecho dimensions are automatically detected during the input preparation and automatically converted to SSA-compatible data. We provide ready-to-use processing scripts and instructions for all of the herein presented pulse sequences, which only require the adjustment of general processing parameters such as apodization or zero filling. All instructions and scripts can be found in the SI and in the data publication associated with this paper (https://data.tu-dortmund.de/previewurl.xhtml?token=2bbfe189-be14-4fa9-8b4f-b25aefc89e82).

### Four-Dimensional Experiments

**Figure 2:**
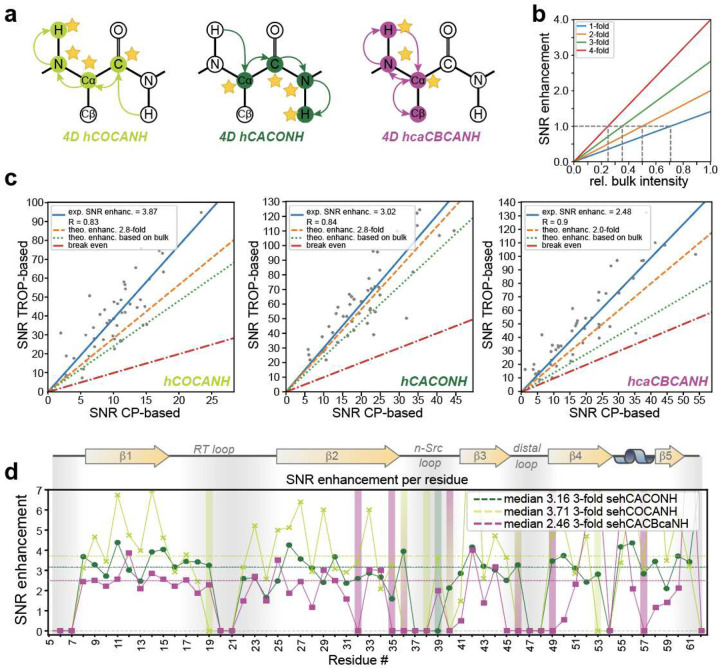
Sensitivity enhancement in H/N/C 4D experiments. **a** Magnetization pathways of the sensitivity-enhanced experiments shown in panels **c** and **d**. Nuclei with a star denote sensitivity-enhanced dimensions (including the directly sampled domain). **b** Obtainable SNR enhancements for different numbers of enhanced indirect dimensions in dependence of the achieved bulk signal relative to the CP-based experiment. The vertical dashed lines indicate the minimum bulk signal (relative to the conventional experiment) that must be achieved to reach at least the same SNR as in conventional experiments. Only for equal bulk signal intensities, the theoretical maximum SNR enhancement is reached (values reached on the extreme right). **c** SNR enhancements for different 4D experiments recorded on a triple-labelled sample of chicken α-spectrin at 55 kHz MAS and a ^1^H Larmor frequency of 800 MHz (hCOCANH, hCACONH) or 500 MHz (hcaCBCANH). Shown are data for the 3-fold enhanced hCOCANH and hCACONH experiments, as well as for the 2-fold enhanced hcaCBCANH. Each plot shows the average experimentally achieved SNR enhancement (solid blue line) along the theoretically obtainable one in case the obtained bulk signal was unchanged (dashed orange line) as well as the theoretical improvement taking the changes in bulk signal into account (dotted green line). The red line indicates the break-even point, where no SNR enhancement would be obtained. **d** Comparison of the residue-specific relative performance (the enhancement of the TROP experiments over CP versions) between the different 4D spectra recorded at either 800 (blue and orange; hCACONH/hCOCANH) or 500 MHz proton Larmor frequency (purple; hcaCBCANH). Colored bars indicate residues that are only visible in the sensitivity-enhanced experiments but cannot be detected in the conventional CP-based version and are therefore excluded from the SNR determination.

To validate these theoretical considerations, we composed a series of 4D and 5D experiments, in which all or part of the indirect dimensions are sensitivity-enhanced via TROPs. Fig. 2 demonstrates the application to 4D hCACONH and hCOCANH experiments, recorded using a micro-crystalline sample of perdeuterated and 100 % amide proton-backexchanged SH3 domain of chicken α-spectrin. The SNR improvement is represented as a two-dimensional correlation to retain residue specificity and visualize the degree of consistency. The experiments were recoded using published TROP shaped pulses downloaded from the optimal-nmr.net website. Three-fold enhanced experiments feature an expected SNR improvement by a factor of ca. 2.8 or time savings by approximately a factor of 7.8. Compared to the CP versions, their actual sensitivity advantage, however, amounts to a factor of ~3.9 or 3.0, respectively. This is even more astonishing, as the bulk signal obtained by the first scans offers sensitivity of only ~80 %, compared to the CP version, which is ascribed to unfavorable properties of NH TROP element initially used here^53^. The higher-than-expected SNR increase upon sensitivity enhancement is remarkable and reflects the non-linear effects of sensitivity for NUS reconstruction (see below). It is noteworthy that the estimation of noise in the case of NUS has often created confusion as artifacts are present through the point spread function that scale with the intensity of peaks present. Hence, noise, as read by processing software CCPNmr^54^ or Poky^55^, is referring to the mixture of sampling artifacts and thermal noise. For the peculiar case of 5Ds, we avoided the noise estimates provided by these software packages as they seem highly biased by the sampled points and instead tailored a SNR estimator for 5Ds, details for which are laid out in the methods section. For this, we specifically interrogate 100 random positions outside a certain radius of a confirmed peak in a given 2D slice of the 5D in an automated fashion. The SNR increase and its non-linearity are also specifically elucidated and confirmed here by comparison of reconstructed and unreconstructed (nuFT) spectra as well the quantification of noise reduction upon reconstruction (see below).

Fig. 2 also includes data for a two-fold enhanced 4D hcaCBCANH experiment, enabling the interrogation of Cβ resonances, required for backbone assignment, and confirming specifically that the framework provides the expected improvements also for *partially* enhanced experiments. (A second example for partial enhancement, the two-fold and three-fold enhanced 5D HNcoCANH will be discussed below.) It should be noted that homoSPEPS^42^ transfers would enable a fully enhanced hcaCBCANH experiment. Similarly, while this manuscript was prepared, also the feasibility of TOCSY^56^ for sensitivity-enhanced transfers and hence hCXCANH-type correlations was shown.^45^

### Five-Dimensional Experiments based on an NCO TROP

**Figure 3:**
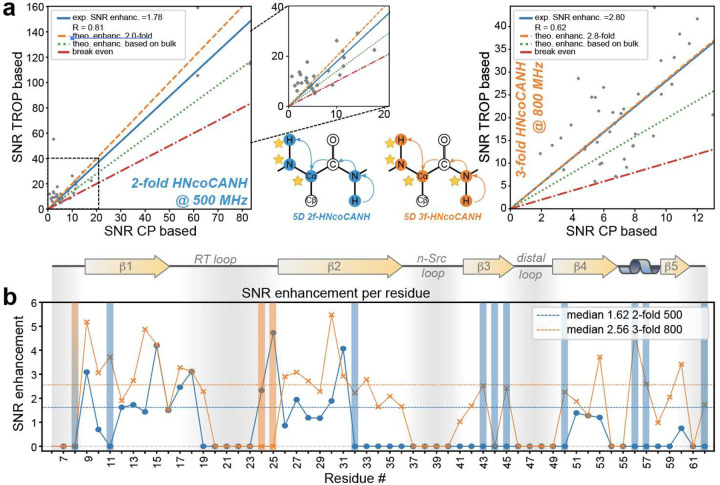
Application of tailor-made TROP pulses to 5D amide-to-amide correlations. **a** SNR enhancements and magnetization pathways of partially-enhanced two-fold and three-fold 5D experiments recorded on the SH3-domain of chicken α-spectrin at different ^1^H Larmor frequencies of 800 MHz (Bruker NEO, right) and 500 MHz (Bruker Avance II, left). Sensitivity-enhanced dimensions are again highlighted by stars, including the direct dimension. Even in NMR experiments that are more challenging, in terms of more complex pulse sequences as well as data processing, the mixing of States-TPPI and E/A dimensions results in the expected SNR gain, further increasing the attractivity of those experiments for backbone resonance assignment for proteins of different sizes, as substantial time-savings or improvements in spectral quality are possible. Details on SNR estimation in 5Ds can be found in the SI. **b** Comparison of the residue-specific relative performance (the enhancement of the TROP experiments over CP versions) between the two different 5Ds. Colored bars indicate residues that are only visible in the sensitivity-enhanced experiments but cannot be detected in the conventional CP-based version and are therefore excluded from the SNR determination.

Assignment of increasingly complex proteins hinges on effective dispersion of otherwise overlapping correlations.^57^ Ways to improve SNR and save measurement time for the associated pulse sequences, whose complexity is intimately coupled to sensitivity hurdles, can thus be vital. For 5D HNcoCANH experiments, on one hand, incorporating published and tested TROPs enables a doubly-enhanced version in which the CAN and NH transfer are sensitivity-enhanced. In addition, we developed and incorporated a new, so far unpublished NCO TROP, in combination with the available COCA homoTROP^53^, which now allows for a triply-enhanced experiment. Figs. 3a and b show the sensitivity enhancements that were obtained after incorporation of two or three enhanced indirect dimensions. The doubly enhanced experiment, recorded at 500 MHz proton Larmor frequency, shows improvements of 1.8, close to the theoretical value of 2 and well beyond the improvements expected when taking the 70 % drop in bulk SNR into account, similar to the 4D results described above (for a discussion of SNR and NUS see below). For these experiments in particular, which for high-molecular weight targets in our hands usually need to be recorded over close to a month, a two-fold SNR increase translates into tremendous time-savings of around two weeks already for the doubly-enhanced option. In the particular SH3 data sets here, recorded time equivalently over ca. 2.5 days on a 500 MHz spectrometer, the sensitivity-enhanced experiment enables the identification of ten more resonances compared to the CP-based experiment (blue or blue-orange bars in Fig. 3b), representative for a resonance assignment process that ultimately in other targets is strongly facilitated. Amide-to-amide (HNCOCANH-type) experiments with more than two enhanced dimensions require new, previously untested TROPs, independent of their dimensionality, such as NCO or HN transfers, both of which have not been applied/created in prior work. The HN transfer has remained challenging due to the underlying ^1^H-density problems^53^, while enhancing the first protein dimension would also require alternative water suppression strategies. The incorporation of the new NCO TROP, however, enables pathways with uninterrupted preservation of complex indirect FIDs from the first N until acquisition (N→CO→CA→N→H). In the specific case of the 5D HNcoCANH, this allows a triply enhanced version, with the associated benefits of even shorter measurement times or better SNRs compared to the doubly enhanced version above. Fig. 3a (right) and b show the remarkable SNR gains close to a factor of 3 after NUS reconstruction, along with additional residues that in this setup are only detected in the sensitivity-enhanced version (solid orange and blue-orange bars). As will be laid out in detail below, further SNR enhancements of NUS experiments are inherently connected to the non-linearity of reconstruction algorithms.

### Robustness of TROP-based sequences

**Figure 4:**
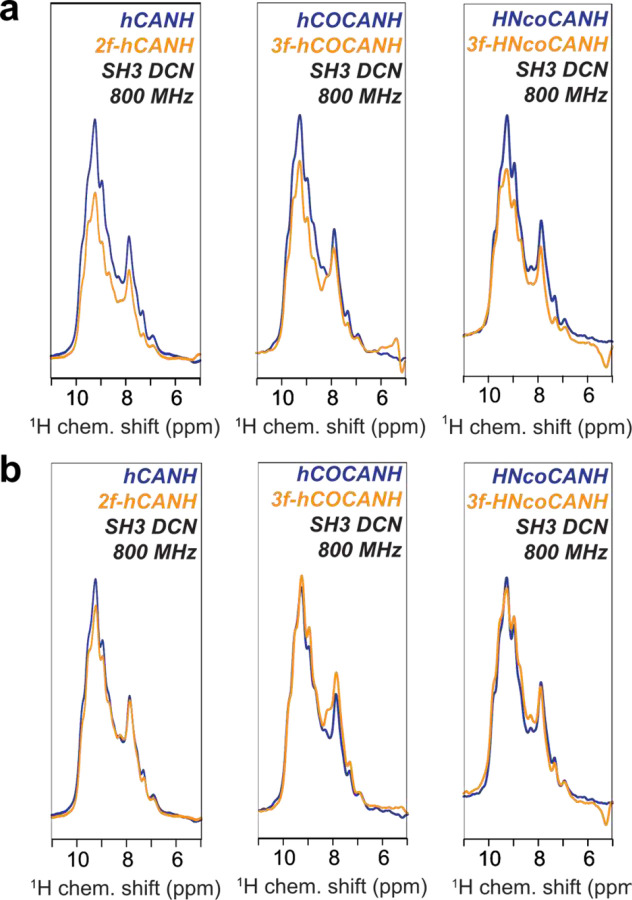
Comparison of bulk sensitivities obtained with either the published and demonstrated NH TROP or a work-in-progress version with improved performance. **a** Sensitivities of the first FID for the triply-enhanced 5D HNcoCANH and 4D hCOCANH experiments as well as the previously described hCANH experiment, each employing the published version of the NH TROP. **b** Comparison of the first FID for the hCANH, hCOCANH and HNcoCANH between CP and doubly/triple-enhanced TROP versions, now using a further improved NH TROP that features improved consideration of varying ^1^H density. In contrast to the earlier NH TROP version used above, the overall sensitivity of the enhanced experiment relative to the respective CP-based one is maintained for each experiment, while the functionality of the respective sensitivity enhancement (in this case the nitrogen dimension) is untouched.

We recorded multiple sensitivity-enhanced experiments for different samples, with different isotope labeling, and on different spectrometers, to identify potential limits or systematic trends in the herein proposed experiments. Fig. 4a and supplementary Fig. 2 show that the relative bulk intensities, measuring the first FID of each experiment relative to the CP-based version, are very similar, irrespective of hardware configuration and sample preparation, for fully enhanced and partially enhanced pulse sequences as well as with varying protonation levels (Supplementary Fig. 2). Supplementary Fig. 1 shows that consistent enhancement factors are gained for the same experiment, demonstrated for the more complex 2-fold enhanced 5D HNcoCANH experiment recorded at 800 MHz. In addition, the bulk intensities shown in supplementary Fig. 2 suggest that the overall sensitivity of the proposed pulse sequences is not substantially different when employing the recently introduced algal extract labelling scheme^58^ using commercial ISOGRO powder (similar results are expected for other commercial amino acid extracts or self-made *E.coli* cell lysates^59^), even though H^α^ and side chain protonation levels are higher compared to perdeuterated sample preparations. (These labeling schemes promise to extract more complete information from the obtained NMR spectra, as ^1^H back exchange problems in the hydrophobic core, a common bottleneck for perdeuterated samples, are avoided.) The combination of these labelling schemes with sensitivity-enhanced pulse sequences hence proves to be viable.

CP transfers are a robust method to transfer magnetization from one nucleus onto another and solely rely on matched resonance conditions. Depending on the desired transfer, e.g., Hartmann-Hahn or HORROR, these conditions are reached by adjusting the rf field strength, with ramps accounting for pulse imperfections, rf inhomogeneities, and other factors, granting robustness irrespective of the spectrometer setup. TROPs, the related tm-SPICE pulses^60^, and generally pulses derived by the OCT module of SIMPSON^61^ aim to overcome these limitations by directly accounting and compensating for them during the optimization, e.g., by considering volume selectivity^62, 63^. As previously reported by Blahut et al.^53^, most TROPs are not susceptible to site-specific differences, but the NH TROP used for the above figures still is. Indeed, a relatively consistent trend along the primary sequences can be identified that both, the 5Ds and the more sensitive 4Ds adhere to (Figs. 2d and 3b).

### A High-Performance NH TROP

Accordingly, considerable effort is currently devoted to improve the general performance of NH TROP transfers, with promising results of using a new, work-in-progress version being demonstrated in Fig. 4. While the written form of this manuscript was almost completed, a new version of NH TROP shapes was generated that are less susceptible to the chemical surrounding. Considering more spins and therefore a locally higher ^1^H density in diverse arrangements, assuming weak as well as strong dipolar couplings, these new shapes can successfully overcome the aforementioned loss of bulk intensity compared to CP-based experiments of around 20–30 %, further augmenting the above sensitivity gains. Detailed description of these optimizations from a technical side is devoted to a forthcoming separate publication, Nevertheless, we consider it important to include the new prospects with these shapes into the current manuscript, as the level of enhancement is further pushed up. Accordingly, as a representative set for the above experiments and generally, bulk intensities for the 4D hCOCANH and 3-fold enhanced 5D HNcoCANH experiments as well as a simple 3D hCANH were recorded at 800 MHz employing the new NH shapes (Fig. 4). In all cases, the bulk signal of the enhanced experiments now is at least on par to the CP-derived signal, i.e., a relative bulk signal of 1.0 for the 5D HNcoCANH and the 3D hCANH, and in case of the 4D hCOCANH even slightly surpassing it with a relative signal intensity of ca. 1.1 (Fig. 4b). With the previous-generation NH TROP, used in the remainder of the manuscript, the consistent loss of 20–30 % that becomes avoidable now is obvious (Fig. 4a). While it would have been unreasonable to rerecord all other data of this manuscript again to also include this new NH TROP, this subset of experiments proves that pulse sequences employing the newest generation of shapes are ultimately capable of fully compensating the mentioned losses associated with a lower bulk signal with no further compromise, warranting a further signal-to-noise enhancement of around 1.3-fold for all of the experiments demonstrated in the remainder of this report, as the pulse was still unavailable at the time they were recorded.

As the data processing workflow is robust once established, representative per-residue SNR enhancements can be directly derived from the signal intensity of the first FID, as these effectively present the only scaling factor (Fig. 2c), supplying the user with a fast and easy method to evaluate the potential performance for their specific configuration and needs. (See NUS reconstruction-specific considerations below.) Table 1 provides an overview for the experiments presented here, summarizing the observed and expected SNR enhancements together with the associated time savings across different labelling schemes and hardware configurations. Even though the SNR gains are naturally more modest for partially enhanced experiments, e.g., compared to the fully enhanced 4D experiments, they still generally imply a factor of two to four in measurement time reduction, meaning tremendous absolute impacts on spectrometer occupation when high-dimensional experiments are recorded for large proteins. In particular for the triply enhanced 4D or 5D experiments, the measurement time reduces to a fraction. Consequently, an entire set of experiments necessary for backbone assignment (assuming for example hCACONH, hCOCANH, and hcaCBCANH) can be recorded in less time than a single CP-based version of one of these experiments would have cost previously.

### Non-Linearity of Sensitivity Enhancement

**Figure 5:**
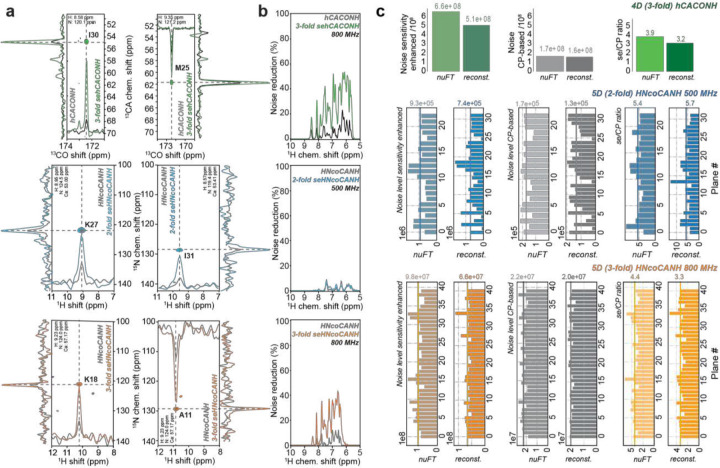
Performance of sampling noise cleaning upon NUS reconstruction. **a** 2D planes from different 4D and 5D experiments recorded with different numbers of sensitivity-enhanced dimensions and at different fields, overlaying the conventional experiments and the experiments based on TROP pulses. Each set of spectra was set to a common noise level to grant comparability. Shown are a fully enhanced 4D hCACONH (top) and two 5D HNcoCANH experiments recorded either as a doubly-enhanced experiment at 500 MHz (middle) or as a triply enhanced version at 800 MHz (bottom). The latter involves the new NCO TROP. **B** Reduction of NUS artifact noise of the corresponding spectra in **a** as returned by SSA after reconstruction. Improved NUS reconstruction contributes to the disproportionate SNR improvement observed especially in the fully enhanced 4D experiments as well as the triply enhanced 5D experiment. Conversely, the comparison of the planes from the doubly enhanced 5D experiment at 500 MHz in the center emphasizes that that the expected SNR improvement is evident even for comparably poor artifact removal (and can therefore not be due to improved sampling artifact removal), but no extra gain is present. **c** Noise levels found in the respective spectra before reconstruction, i.e., applying only nuFT (the left one of each pair of columns), and after NUS reconstruction with SSA (right column of each pair). Shown are levels for the sensitivity-enhanced spectra (left pair) and CP-based spectra (center pair) as well as their ratios (right pair), whereby for improved understandability of the data for the top row (the hCACONH) only the average numbers over all peaks are shown and the 5Ds are depicted plane-specifically, the resulting average numbers being denoted by a vertical line and an annotation above each graph.

We wondered how gains higher than the theoretical maximum (taking respective relative bulk sensitivities into account) can be rationalized. As stated above, acquisition and reconstruction of NUS spectra is effectively required when spectra of four or more dimensions are recorded. Nowadays, there is a plethora of algorithms that can routinely handle 4D spectra. Reconstruction of 5D experiments is associated with a limited number of options, and dimensionality > 5D is limited to more exotic types of data processing to-date^64, 65^. SMILE^52^, hmsIST^51^, and SSA^50^ represent a selection of the available algorithms for 4D reconstruction, while in our hands only SSA is employed routinely for the reconstruction of 5D experiments.^66^ While all the algorithms follow different strategies to remove sampling artifacts from the spectrum, they all more or less rely on a sufficient SNR per peak to distinguish between signals and sampling artifacts. As shown in Fig. 5, the suppression of sampling noise in fully enhanced 4D experiments as well as for the triply enhanced 5D HNcoCANH becomes more than twice as effective as for the conventionally recorded data sets (top and bottom row), reaching levels that in our hands are often not even reached for the reconstruction of CP-based 3D experiments. Differentially successful reconstruction of these spectra also explains the over-proportional SNR enhancements (blue graphs compared to the green dotted graphs in Figs. 2 and 3) found for the hCOCANH and hCACONH experiments shown in Fig. 2a, as well as for the three-fold enhanced 5D (Fig. 3a,b and Fig. 5, bottom row). (Note that the additional gain upon reconstruction of higher-SNR data is not to be expected upon comparison of individual (strong vs. weak) peaks within a single spectrum (e.g., regarding the differential intensities depicted in the correlations in Figs. 2c and 3a), as improved removal of convoluted sampling artifacts due to the point spread function improves to the *entire* spectrum.) By contrast, the improvement of the SNR in the absence of reconstruction (upon nuFT) tends to be very close to the theoretical values expected taking the bulk signal into account. (Supplementary Fig. 3 and 4 include enhancement factors found for the triply enhanced and doubly enhanced 5D experiments before reconstruction, i.e., using nuFT.) Whereas Fig. 5a/b show the obtained spectra and the success of artifact removal, respectively, Fig. 5c specifically compares the noise levels before (nuFT) and after reconstruction of the different NUS spectra more quantitatively. The effective noise level consistently reduces upon reconstruction, as this noise includes sampling-based artifacts, which are irradicated in the process of reconstruction. Importantly, this artifact reduction is stronger/more effective for the enhanced spectra, such that the comparison between the noise levels in the sensitivity-enhanced vs. the CP-based variants and hence the difference in SNR becomes even more favorable than in the US case (represented by nuFT). The sensitivity-enhanced spectra feature an overall higher noise level, as the artifact level derives from convolution and is proportional to the intensity of the actual peaks, such that the ratio sensitivity-enhanced/CP for the noise should always be greater than 1. (Note that the final sensitivity is the signal-to-noise ratio, and the increase in absolute noise levels is compensated by an increase in sensitivity, which is not the scope of the comparison here.) Compared to the nuFT data, the reference point reflecting the “pure”/original increase in sampling noise, reconstruction is now able to eliminate the artifacts, which procedure works better for higher signal intensity (i.e., for the sensitivity-enhanced experiments). Accordingly, after reconstruction, the noise in the sensitivity-enhanced spectra is increased compared to the CP data but less than in the nuFT case. Comparison of the noise (ratio sensitivity-enhanced vs. CP spectra) after reconstruction hence hence yields much smaller numbers than before reconstruction (rightmost column in Fig. 5). The more effective noise reduction/SNR improvement achieved by sensitivity enhancement, i.e., an increased sensitivity in a given transfer, can hence translate into over-proportionally higher sensitivity enhancement of the overall reconstructed data sets. The example in Fig. 5 center row, however, also shows that for spectra of very low overall SNR, these additional increases in effective sensitivity can be virtually absent, and the obtained sensitivity enhancement is limited to the actual PEP effects only. In conclusion, with NUS, the artifact noise level depends, among other factors, on the intensity of the signals^67^, whereas thermal noise is uncorrelated of such. Expressed in a more condensed form, nuFT spectra of sensitivity-enhanced experiments initially feature both, higher-intensity peaks and a high noise level compared to conventional spectra, however, artifact removal during reconstruction and hence reconstruction of the clean spectrum are facilitated as more signals can be identified more faithfully, and extra gains can be obtained.

The above data demonstrate important prospects of sensitivity enhancement for protein resonance assignment. Particularly when target complexity demands more sophisticated assignment sequences to disperse overlap into higher-dimensional chemical-shift space, the concatenated use of multiple TROPs translates into a major sensitivity boost. Such a boost on the sensitivity side is usually linked to a trickle-down effect through the entire NMR processing pipeline, including peak picking (manual and automated) being less ambiguous, the assignment process being alleviated, and downstream applications, such as relaxation series, being successfully completed earlier. Challenging preparations, dilution of membrane proteins by a lipid embedding, or broader line widths have not disqualified the use of NUS but tended to entail less successful NUS reconstructions, i.e., in particular, incomplete data sets even at the highest B_0_ fields available to date. This is further aggravated for proteins of higher molecular weight, where the nominal SNR decreases linearly with the increase in molecular size. We hence anticipate that preservation of equivalent pathways in multiple dimensions will be transformative for the concurrent molecular-weight limits, which are closely associated with the sensitivity of more sophisticated pulse sequences for sufficient dispersion/assignment fidelity. The inherent non-linearity of NUS reconstruction, plainly speaking, the bottleneck of a sufficient signal to noise ratio to successfully identify a peak as such, has been aggravating the intricate relationship between sample properties and NMR accessibility. Accordingly, for many systems sensitivity-enhanced experiments will be decisive regarding whether higher-dimensional spectra and thus their overall NMR assessment is realistically possible within the existing resources of measurement time. Due to the translation of the effective SNR improvements of reconstructed NUS data into time saving with a quadratic relationship, the reported effect of sensitivity enhancement in higher-dimensional spectra, enabling a decrease of measurement time of up to 10-fold, can be expected to be game-changing for solid-state NMR assessment for a large range of samples. Importantly, a factor of 10 in time needed should not be understood as a mere „saving” of measurement time or an improved efficacy of any solid-state NMR infrastructure. Instead, those solid-state NMR experiments in particular that are critical for competitive biological targets will only be enabled by the presented technology, which conceptually redefines the feasibility of solid-state NMR and its impact in structural biology.

## CONCLUSION

Here, we have shown the use and tremendous benefits of a set of tailor-made optimal-control pulses (TROPs) for sensitivity-enhanced magnetization transfer in higher-dimensional (4D and 5D) spectra, which are crucial to grant NMR resonance assignment in protein targets of increasing size and complexity. Apart from demonstrating the flexibility and robustness of TROPs across various conditions, including different probes, consoles and sample preparations, the proposed experiments, in conjunction with the framework conditions of reconstruction of non-uniformly sampled data, are shown to enable time savings up to an order of magnitude. Together with the tailor-made processing tools and pulse sequences required for their widespread implementation provided, we expect a transformative impact of these strategies on the feasibility of solid-state NMR studies for a multitude of samples that have been escaping interrogation till-date due to high molecular-weight or inhomogeneity.

## METHODS

### Sample Preparation

The uniformly [^2^H,^13^C,^15^N]-labelled SH3 domain of chicken α-spectrin was obtained through recombinant protein expression in *E.coli* BL21(DE3) cells using ^15^NH_4_Cl and u-[^2^H,^13^C] glucose in D_2_O-based M9 medium. A 100% back-exchanged sample was obtained through purification in aqueous buffer and consecutive micro-crystallization through addition of (NH_4_)_2_SO_4_, final concentration 100 mM, and a pH shift from pH 3.5 to pH 8. To accelerate data acquisition by PRE, 75 mM Cu(EDTA), final concentration, was added during crystallization^68, 69^.

A sample of tryptophan synthase (TS) of *S. typhimurium* using the algal extract labelling scheme with commercial ISOGRO powder was obtained through recombinant expression in *E.coli* CB149 (TS knock out) cells in H_2_O based M9 medium using solely ISOGRO as a sole growth medium^58^. Further purification and crystallization followed previously described protocols^58, 70^. Paramagnetic doping was achieved by addition of 20 mM Cu(EDTA), final concentration, to the microcrystals and incubation overnight before rotor filling.

The respective rotors were filled by centrifugation of the microcrystalline samples into 1.3 mm rotors containing fluorinated rubber plugs.

### NMR Spectroscopy

All experiments were recorded using a HNC triple resonance MAS solid-state probe spinning a 1.3 mm rotor at 55.555 kHz at different magnetic fields. The effective sample temperature was ca. 25 C in all cases. Acquisition parameters and RF field strength for the respective experiments are listed in the SI.

### SNR Estimation in 5D Spectra

To estimate the SNR of individual signals in 5D spectra processed as stacks of 2D planes as done by SMFT, we calculated a SNR for each individual plane, i.e., each individual peak rather than using the globally estimated value suggested by common NMR software packages such as CCPNmr or Poky as those have shown large variations in the estimated noise level for every new calculation. Our estimation employes a rudimentary but robust method to measure the noise level for every plane, by automatically picking 100 random points while excluding an area of ± 1.0 ppm along the indirect ^1^H_i+1_ dimension and ± 2.5 ppm along the ^15^N_i+1_ dimension to avoid extraordinarily high peak intensities biasing the estimation. Before SNR calculation, the mean noise level was subtracted from the peak intensities, virtually enforcing a mean noise level of 0. The noise level was then calculated as the standard deviation, and SNR calculated as peak intensity / noise level.

## Supplementary Files

This is a list of supplementary files associated with this preprint. Click to download.

- SI.pdf

## Figures and Tables

**Table 1:** Measurement-time equivalents to reach the same SNR compared to the same experiment based on CPs only and compared to an hNH, both recorded on the same sample. Numbers are based on the expected SNR enhancements based on bulk signal, including the measured relative signal of the first scan and the theoretical gain derived from the preservation of equivalent pathways for a given number of enhanced indirect dimensions.

Type (# enh. dim.)	Bulk^a^		Field (MHz)	sample	SNR enhancement^a^		Time equivalents (based on expect.)^a^	
	*% of hNH*	*rel. to conv. (old/new NH)*			*expected*	*observed*	*of conv*.	*to hNH SNR*
*hCOCANH (3)*	15 / *19*	0.85 / *1.1*	800	SH3 DCN	2.4 / *3.1*	3.9 *(5.0)*	0.17 / *0.1*	31 / *18*
*hCACONH (3)*	15 *(20)*	0.85 *(1.1)*	800	SH3 DCN	2.4	3.0 *(4.1)*	0.17 (0.1*)	31 (18*)
	15 *(20)*	0.7 *(1.0)*	700	TS ISO	2.0		0.26	45
*hcaCBCAN H (2)*	8 *(11)*	0.8 *(1.0)*	500	SH3 DCN	1.6	2.5 *(3.4)*	0.39 (0.21*)	240 (130*)
	8 *(11)*	0.7 *(1.0)*	700	TS ISO	1.4		0.51	319
								
*HNcoCANH (2)*	7.5 *(10)*	0.87 *(1.1)*	800	SH3 DCN	1.7	1.8 *(2.4)*	0.33 (0.18*)	470 (260*)
*HNcoCANH (2)*	7.5 *(10)*	0.7 *(1.0)*	500	SH3 DCN	1.4	1.4 *(1.9)*	0.51 (0.28*)	730 (400*)
*HNcoCANH (3)*	7.5 / *11*	0.7 / *1.0*	800	SH3 DCN	2.0/ *2.8*	2.7 *(3.9)*	0.26 / *0.13*	360 / *180*

a For the hCOCANH and 3f-HNcoCANH experiments (first and last row), the bulk signal was additionally determined with an improved NH TROP (“new NH”), which improves the bulk signal by 1.3 and 1.45 fold over the published NH TROP, respectively (numbers in italics). For the other rows, the additional values (then in brackets) reflect values also taking a respective bulk signal improvement of 1.35 into account that would have been attained here, too, if these newest-generation NH TROPs had already been available.

## Data Availability

Commented pulse programs in Bruker format, NUS processing scripts and step-by-step instructions for the respective experiments are supplied in the SI and can be downloaded from the respective data publication (https://data.tu-dortmund.de/previewurl.xhtml?token=2bbfe189-be14-4fa9-8b4f-b25aefc89e82) The data publication further includes raw data in Bruker format (ser files), all necessary processing scripts, and either fully reconstructed spectra or projections of those. All TROP shapes except the updated NH TROP can be downloaded under www.optimal-nmr.net.
